# Fixed-Bed Column Adsorption Studies: Comparison of Alginate-Based Adsorbents for La(III) Ions Recovery

**DOI:** 10.3390/ma16031058

**Published:** 2023-01-25

**Authors:** Dominika Fila, Dorota Kołodyńska

**Affiliations:** Department of Inorganic Chemistry, Institute of Chemical Sciences, Faculty of Chemistry, Maria Curie-Skłodowska University, Maria Curie-Skłodowska Sq. 2, 20-031 Lublin, Poland

**Keywords:** alginate-based adsorbents, lanthanum, column studies, desorption

## Abstract

The paper investigated the adsorption of the packed-bed column with the alginate-based adsorbents (ALG-based adsorbents) such as alginate-biochar, alginate-clinoptilolite, alginate-lignin, and alginate-cellulose for La(III) ions’ removal. Fixed-bed adsorption studies with various alginate-based adsorbents were carried out and compared to the La(III) ions adsorption. The columns were filled with ALG-based adsorbent beads of approximately 1.1 ± 0.005 mm spherical shapes. The effects of the inlet concentrations on the breakthrough curves were studied in terms of the adsorption performance of the ALG-based adsorbents. The experimental data were correlated with the Adams-Bohart, Yoon-Nelson, Thomas, and Wolborska models to determine the best operational parameters. Based on the comparison of R^2^ values, the Thomas and Yoon-Nelson models were found to be more suitable than the Adams–Bohart and Wolborska models. In the desorption study, the ALG-based adsorbents packed columns showed the maximum desorption of La(III) just after passing 100 cm^3^ of 1 mol/dm^3^ HCl. Overall, the results show that ALG-based adsorbents could be used for continuous recovery of La(III) ions from aqueous solutions and were not only cost-effective but also environmentally friendly.

## 1. Introduction

Strategic elements, such as rare earth elements (REEs), play a critical role in industry, especially in the production of high-tech materials. Major global industries developed a strong dependence on rare earth materials. Every year, innovations appear in sectors, such as modern technology, green energy, or communication technologies, requiring more strategic metals to increase investment profitability [1,2,3,4,5]. REEs were also applied in medicine for cancer imaging and therapy [6]. The increasing demand for rare earth elements due to their exponential use in various applications has stimulated research on the development of an efficient technology for their separation and recovery.

**Figure 1:**
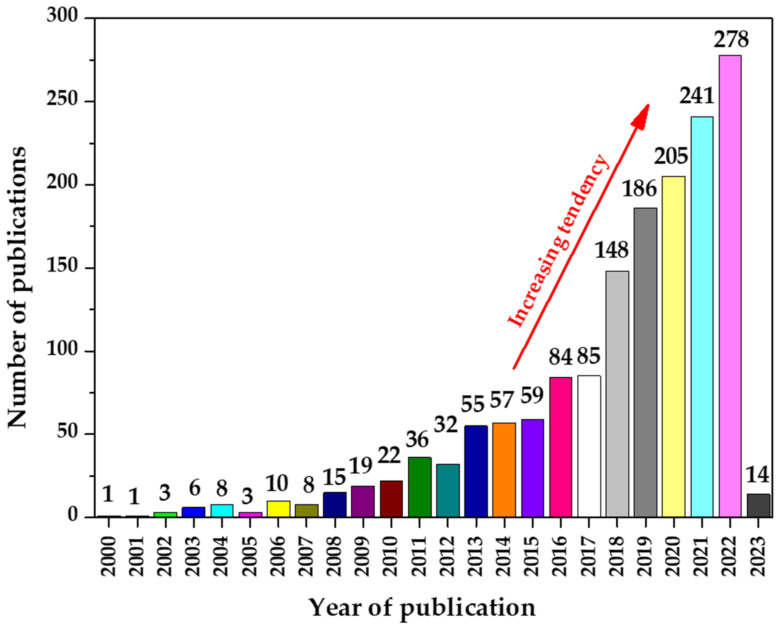
Number of publications with the words “alginate adsorbents” based on the Scopus database score (data from 1 December 2022).

Due to the availability of a broad spectrum of both natural and artificial adsorbents, the adsorption method is a very promising technology. Zeolite [7], charcoal [8], metal oxides [9], carbon nanostructures [10], activated carbon [11], and natural polymers [12] are significant adsorbents. Among them, alginate adsorbents stand out due to several advantageous features such as low-cost, easy production, and availability [13]. The increasing trend of publications with the words “alginate adsorbents”, as can be seen in Figure 1, supports the significance of alginate.

Alginate has hydroxyl and carboxyl groups on its surface, and these groups have a great attraction for radionuclides [14] as well as heavy metal ions [15,16]. However, occasionally, their insufficient physical characteristics, as well as water solubility, limit their potential usage. By changing their surface and mixing them with other materials, these limitations can be somewhat circumvented. Therefore, investigating alginate derivatives by surface functionalization of the polymer has always been a fascinating issue for researchers, with the aim of enhancing its mechanical strength and stability for environmental applications. The literature data results showed that the ability to adsorb was enhanced by adding extra components to natural polymers. For example, alginate beads were modified with sodium polyacrylate for Pb(II) removal [17], with chitosan from shrimp shells for Cd(II) removal [18], with bentonite for phosphate removal [19], with chitosan and TiO_2_ for Ni(II) removal [20], with graphene oxide for Pb(II), Zn(II), and Cd(II) removal [21]**,** as well as with sericin and polyvinyl alcohol for La(III) recovery [22]. Chemical modification by grafting of urea and biuret on the alginate backbone increases by 53 to 84% the sorption efficiency of the alginate for Cd(II), Cu(II) and Pb(II) recovery [23].

For the preliminary research on an adsorbent material, batch system tests are used since they reveal the maximum amount that can be adsorbed by a given unit of adsorbent as well as the efficacy of the adsorption for removing particular impurities. The batch mode, however, is restricted to treating small amounts of effluent and does not offer information on the precise dimensions of continuous treatment systems. Because of their simplicity and high removal effectiveness, the continuous adsorption procedures in the fixed-bed columns are typically used in industrial applications to treat wastewater on a large scale. The fixed-bed sorption system establishes the adsorbent practical applicability in the continuous mode, which is also appropriate for large scale wastewater volumes [24,25]. The removal of metal ions by adsorption using a column system is very advantageous due to the column system adaptation to versatile processes, less reagent control, and low operational cost [26,27]. The most common column studies onto alginate-based materials were carried out for heavy metal ions such as Cu(II) [28], Cd(II) [29], Pb(II) [26,30], Cr(III) [31], Cr(VI) [32], as well as dyes such as methylene blue [33], congo red [34], and red [35]. There is currently a gap in the literature regarding the column mode adsorption studies with the alginate adsorbents, particularly in the adsorption of REEs. Thus, in this paper, the applicability of alginate-based adsorbents in the column system for La(III) adsorption was investigated. The effect of the initial La(III) ion concentration on the breakthrough curves was studied. For possible extension of the fixed-bed column from the laboratory scale to the industrial scale, mathematical modeling was applied. Several mathematical models can be used to describe the profile of concentration vs. time of the column, including the Adams-Bohart, Thomas, Yoon-Nelson, and Wolborska models.

## 2. Experimental Section

### 2.1. Materials

For this study, we used the following materials and reagents: alginic acid sodium salt (1% solution viscosity 0.35–0.55 Pa·s and molar mass: 3–3.5 × 10^5^ g/mol) supplied by Carl Roth (Karlsruhe, Germany), metal hexahydrate salt-like lanthanum(III) nitrate (La(NO_3_)_3_·6H_2_O), the degree of purity at 99.9%, purchased from Sigma Aldrich (St. Louis, Mo, USA), calcium chloride hexahydrate (CaCl_2_·6H_2_O, analytically pure) was obtained from Chempur (Piekary Śląskie, Poland), silver nitrate was supplied by Sigma Aldrich (St. Louis, Mo, USA), and hydrochloric acid (35–38%) and nitric acid (65%) were purchased from Chempur (Piekary Śląskie, Poland). In the column adsorption studies the deionized water of the resistivity about 0.05 µS/cm was also used.

#### Alginate-Based Adsorbents

**Figure 2:**
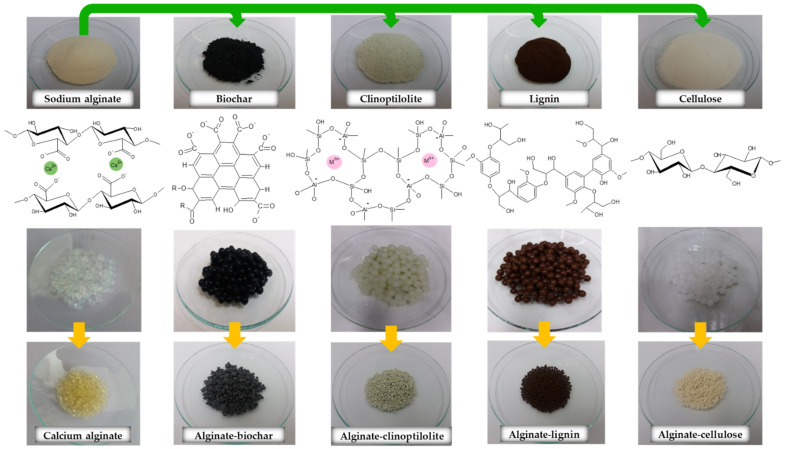
Images of the obtained calcium alginate and alginate-based composites.

The alginate-based adsorbents containing biochar, clinoptilolite, lignin, and cellulose, denoted as ALG-BC, ALG-CPL, ALG-L, and ALG-C, were fabricated using the procedure described in previous papers [36,37]. Briefly, sodium alginate was used as a main component of the synthetized composites but biochar, clinoptilolite, lignin, and cellulose were used as additives. The alginate composites contained 20% additives. The obtained alginate-based composites in the wet and dried form beads are illustrated in Figure 2. Depending on the additive used for the alginate composite production, the composite beads ranging from white to black were obtained.

### 2.2. Methods

#### 2.2.1. Physicochemical Analyses of Alginate-based Adsorbents

The point of zero charge (pH_pzc_) of the synthetized alginate adsorbents was identified using the well-known drift approach detailed in the literature [38]. Next, using nitrogen gas at 77 K, the Autosorb iQ Analyzer (Quantachrome, Graz, Austria) assessed the surface characteristics of the alginate adsorbents. Over a 24-hour period, the samples were degassed at 353 K. The precise surface area was calculated using the conventional BET approach. The pore size distribution was then calculated using the BJH method. With the step size of 0.01° and the range of 4 ≥ 2θ ≤ 60°, the phase compositions of the samples were analyzed using the powder X-ray diffractometer (XRD, AXS D8 Advance diffractometer, Bruker) and Cu Kα radiation (λCu Kα1 = 1.5406 Å). ATR/FT-IR spectroscopy using Agilent Cary 630 FT-IR spectrometer with the ATR accessory (Agilent Technologies, Inc., USA) was performed at room temperature in a range between 500 and 4000 cm^–1^ to detect any functional groups present in the polymers matrix. Measuring the binding energies of the photoelectrons, X-ray photo-electron spectroscopy (XPS) using the UHV Analytical System XPS Multichamber (Specs, Berlin, Germany) was used to detect all elements and perform a quantitative analysis of the surface composition of alginate adsorbents. The X-ray measurements were conducted with a monochromatic source (Al anode). During the analysis the vacuum in the chamber was 1 × 10^−9^ mbar. The CasaXPS application was used to process the obtained data. The thermogravimetry (TG) examination was carried out using the thermal analyzer (Setsys 1200, Setaram, Caluire-et-Cuire, France) with the heating rate of 10 °C per minute to assess the thermal behavior of alginate adsorbents. The temperature measurements in the N_2_ atmosphere ranged from 20 to 1000 °C. The laboratory sieves (Retsch, Katowice, Poland) were used for the sieve analysis (also known as the gradation tests) to determine the particle size distribution in the range from 900 to 1200 µm. The optical microscopy (OM) images were recorded (AZ100M, Nikon Multizoom, Tokyo, Japan) to investigate the morphologies and shape of the ALG-based adsorbents.

#### 2.2.2. Column Experiments Procedure

**Figure 3:**
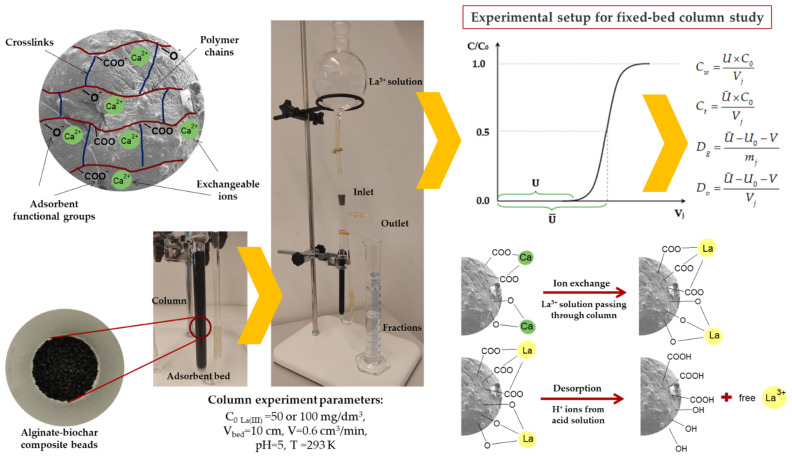
Fixed-bed adsorption column schematic for an experimental study.

The column studies were carried out with the solutions containing 50 and 100 mg/dm^3^ of La(III) ions at pH 5.00 ± 0.05. The choice of pH values for the column tests was dictated by the results obtained earlier from the static tests which were published in the papers [36,37]. At pH 5, the efficiency of the process was the highest and equaled nearly 100%. Thus, the selected pH of the solution was adjusted with either 1 mol/dm^3^ HNO_3_ or 1 mol/dm^3^ NaOH. These studies were carried out at room temperature about 293 K. For the column experiments, the ion exchange glass columns with a 1 cm internal diameter and a 25 cm height were used. Thus, in the glass columns 10 cm^3^ of swollen alginate-based adsorbents were packed. These swollen adsorbents volume corresponded to their dry mass of 3.5 ± 0.25 g and the effective depth of 11.5±0.5 cm. Then, the distilled water was passed through the column. Finally, the La(III) ions solution with the known initial concentration (50 mg/dm^3^ or 100 mg/dm^3^) was passed through the prepared bed at the flow rate of 0.6 cm^3^/min. The eluate was collected into the defined volume fractions and the Inductively coupled plasma optical emission spectrometry (ICP-OES) was used to determine the content of the tested metal ions. The column process was repeated until the concentration of La(III) ions in the effluent equaled the concentration of the solution introduced into the column (50 or 100 mg/dm^3^). When the columns were exhausted, lanthanum(III) ions were desorbed from the adsorbent beds by passing 1 mol/dm^3^ HCl through the column bed at the flow rate of 0.6 cm^3^/min. The procedure was repeated until the concentration of La(III) ions in the eluate was zero. The experimental setup for the column experiments is illustrated in Figure 3.

#### 2.2.3. Column Experiments Calculations

The column experiment results are shown as the breakthrough curves as a function of the eluate volume C/C_0_ = f(V) where C is the concentration of La(III) ions in the effluent [mg/dm^3^], C_0_ is the concentration of La(III) ions in the stock solution [mg/dm^3^], and V is the eluate volume. The basic parameters characterizing the column adsorption studies, such as the adsorption capacities (q_ec_) [mg/g], working (C_w_) and total (C_t_) exchange capacities [mg/cm^3^], mass (D_g_) and volumetric (D_v_) distribution coefficients were calculated using the obtained breakthrough curves:

(1) $$q_{ec}=\frac{U\times C_{0}\times C_{ec}}{m_{j}}$$

(2) $$C_{w}=\frac{U\times C_{0}}{V_{j}}$$

(3) $$C_{t}=\frac{\bar{U}\times C_{0}}{V_{j}}$$

(4) $$D_{g}=\frac{\bar{U}-U_{0}-V}{m_{j}}$$

(5) $$D_{v}=\frac{\bar{U}-U_{0}-V}{V_{j}}$$

where U is the total leakage volume until the breakthrough point [cm^3^], C_0_ and C_ec_ are the initial and equilibrium (at breakthrough) La(III) ions concentrations [mg/dm^3^], m_j_ is the dry bed mass [g], Ū is the leakage volume corresponding to C/C_0_ = 0.5 [cm^3^], V_j_ is the ALG-based adsorbents bed volume placed in the columns [cm^3^], U_0_ is the dead column volume (i.e., the volume of liquid in the column between the bottom edge of the bed and the outlet of the column) [cm^3^], and V is the empty space volume between the ALG-based adsorbents beads (i.e., 0.4 cm^3^).

By integration along the curve, the total ion exchange capacities (C_t integration_) were also determined and compared with the total ion exchange capacities (C_t_) calculated from Equation (3).

#### 2.2.4. Mathematical Models in the Fixed-bed Column Studies

In this study, various models including the Adams-Bohart, Thomas, Yoon-Nelson, and Wolborska models were used to evaluate the behavior of the adsorbent-adsorbate system and to analyze the kinetic model parameters at different runs. The kinetic constants of the above models were calculated using the linear regression analysis.

##### Adams-Bohart Model

The Adams-Bohart (AB) model was first developed in 1920 by G. S. Bohart and E. Q. Adam for the chlorine adsorption from air on activated carbon [39]. The overall isotherm and the correlation between the changing effluent concentrations and the initial concentration are explained by this model. The following linear Equation (6) represents the Adams-Bohart model, which posits that the rate of adsorption is proportional to the concentration of the adsorbed species and the residual capacity of the adsorbent [40].

(6) $$\ln(\frac{C}{C_{0}})=k_{AB}C_{0}t-k_{AB}q_{AB}\frac{H}{\nu }$$

where C_0_ is the metal ions concentration entering the column [mg/dm^3^] and C is the metal ions concentration flowing out of the column [mg/dm^3^], H is the bed height [cm], υ is the linear flow rate calculated by dividing the flow rate by the cross-sectional area of the column [cm/min], k_AB_ is the Adams-Bohart rate constant [dm^3^/mg·min] and q_AB_ is the adsorption capacity per unit volume of the adsorbent bed [mg/dm^3^].

The model parameters k_AB_ and q_AB_ are typically estimated using the linear regression of ln(C/C_0_) vs. t, with only experimental points near the breakthrough point used to describe the initial curve part. Similarly, the AB model nonlinear regression can be also used to describe the entire breakthrough profile.

##### Thomas Model

The Thomas model developed by Henry C. Thomas [41] assumes that the plug flow is observed in the bed. Moreover, this model uses the Langmuir isotherm for the adsorption-desorption equilibrium and the reversible pseudo-second order reaction kinetics. One of the most popular models in the column performance theory and the forecasting breakthrough curves is this one. It is used to calculate an adsorbent maximum capacity. Using the data from the continuous mode experiments in the columns and the Thomas kinetic model, the maximum solid phase concentration of the adsorbate (La(III) ions) onto the adsorbent, and the adsorption rate constant were calculated [42]. Equation (7) illustrates the Thomas model formula for the adsorption column:

(7) $$\ln(\frac{C_{0}}{C}-1)=\frac{k_{Th}q_{0}m}{Q}-\frac{k_{Th}C_{0}}{Q}V$$

where V is the volume of effluent [dm^3^], and Q is the flow rate [cm^3^/min], m is the adsorbent mass in the column bed [g], q_0_ is the maximum adsorption capacity [mg/g], and k_Th_ is the Thomas rate constant [cm^3^/mg·min].

The main parameters of this model were determined from the slope and intercept of the linear plots. The model parameters k_Th_ and q_0_ are typically calculated using the linear regression of ln[(C/C_0_)-1] vs. t.

##### Yoon-Nelson Model

The Yoon-Nelson (YN) model was developed in 1984 by Young H. Yoon and James H. Nelson [43] and focuses on adsorption and vapor or gas breakthrough of adsorbate with respect to activated carbon. The Yoon and Nelson model is based on the assumption that the chance of adsorbate adsorption and breakthrough onto the adsorbent decreases proportionately to the rate of adsorption probability for each adsorbate molecule. This model is less complicated than others because it does not demand specific information regarding the adsorbate properties, the type of adsorbent, and the adsorption physical features. The Yoon and Nelson Equation (8) has the following form:

(8) $$\ln(\frac{C}{C_{0}-C})=(k_{YN}t-k_{YN}\tau )$$

where t is the time [min], τ is the time required to achieve 50% breakthrough time [min], and k_YN_ is the Yoon-Nelson rate constant [1/min].

According to Equation (8), the Yoon-Nelson model plot is comprised of C/(C_0_–C) vs. sampling time (t). The values k_YN_ and τ were obtained using the linear regressive model [17].

##### Wolborska Model

The Wolborska model was developed based on the analysis of p-nitrophenol adsorption on activated carbon [44]. The breakthrough curve in the low concentration range is described by this model. Equations (9)–(10) illustrate the Wolborska model formula:

(9) $$\ln(\frac{C}{C_{0}})=\frac{\beta_{a}C_{0}}{q}t-\frac{\beta_{a}H}{\nu }$$

(10) $$\beta_{a}=\frac{\nu^{2}}{2D}(\sqrt{1+\frac{4\beta_{0}D}{\nu^{2}}}-1)$$

where βa is the external mass transfer kinetic coefficient [1/min], q is the maximum amount of adsorbent that can be absorbed per unit volume [mg/dm^3^], β_0_ is the external mass transfer coefficient with a negligible axial dispersion coefficient, and D is the axial diffusion coefficient [cm^2^/min].

From the plot ln (C/C_0_) vs. t, q and β_a_ can be calculated from the slope and intercept, respectively. This model can become the Bohart–Adams model if k_AB_ = β_a_/q. It frequently only offers a good fit to the first part of the breakthrough curve.

## 3. Results and Discussion

### 3.1. Comparison of Alginate-based Adsorbents Properties

**Figure 4:**
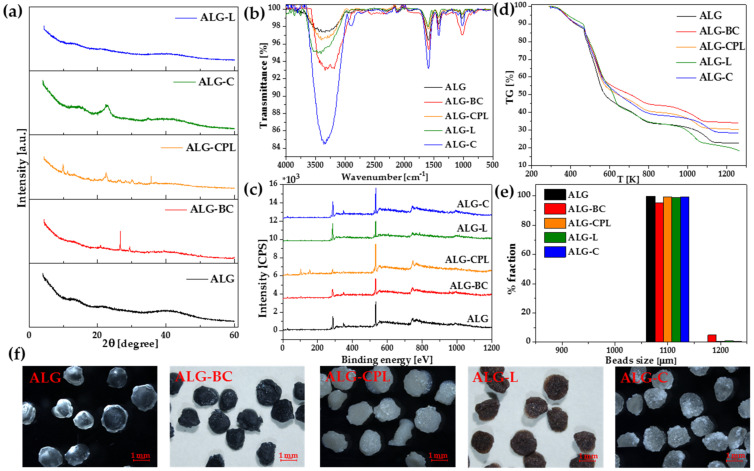
Physicochemical analyses of alginate-based adsorbents. (**a**) XRD pattern, (**b**) ATR/FT-IR spectra, (**c**) XPS spectra, (**d**) TG curves, (**e**) gradation tests, and (**f**) OM images (the scale bar denotes 1 mm length).

The alginate-based adsorbents as beads were fabricated by embedding different additives, i.e., biochar, clinoptilolite, lignin, and cellulose into the cross-linked alginate matrix. The CaCl_2_ solution was used to form the insoluble gel spheres. The studied physicochemical properties of alginate adsorbents are presented in Table 1 and Figure 4.

The addition of additives to the alginate matrix contributed to the introduction of new functional groups into its structure which in turn, translated into a change in its properties. The obtained pH_pzc_ values of raw alginate and alginate modified by biochar, clinoptilolite, lignin, and cellulose were different. The addition of biochar to the alginate matrix resulted in the highest change in the value of pH_pzc_, changing from 6.14 to 7.68. The pH_pzc_ measurements were also determined for raw biochar, clinoptilolite, lignin, and cellulose and the values were 9.64, 6.18, 5.76, and 6.24, respectively.

Generally, biosorbents have low specific surface area. The freshwater algae-based biosorbents showed specific surface areas in the range of 0.6–1.8 m^2^/g [45]. Unmodified plant leaf biosorbents had also low specific surface areas ranging from 0.3 to 20.5 m^2^/g, but their modifications contribute to an increase in their values [46]. The surface area value determined using the BET theory for the raw alginate was low at 4.7 m^2^/g. Modification of the alginate with the biochar, clinoptilolite, lignin, and cellulose additives (Table 1) helped increase its specific surface area to the maximum of 9.8 m^2^/g (the total pore volume also increased). Despite the limitations associated with the relatively low specific surface area of biosorbents, these materials exhibit quite high adsorption capacities. This is explained by the idea that when biosorption is involved, the surface area size value may not be as significant as it first appears. This is possible due to the presence of a huge number of functional groups in the alginate biosorbents’ structure.

Using the X-ray diffractometry method, the crystal structures of alginate, alginate-biochar, alginate-clinoptilolite, alginate-lignin, and alginate-cellulose composites were examined. The biosorbents XRD profile exhibits typical diffraction peaks (Figure 4a). For ALG, ALG-L, and ALG-C broad peaks rather than acute peaks were seen, indicating that the material was not sufficiently crystalline. The X-ray pattern of the alginate-cellulose exhibits the typical diffraction peaks of the crystalline structure of cellulose at 2θ = 14.82 and 22.61° [47]. In the case of ALG-BC and ALG-CPL adsorbents, the characteristic peaks for the biochar and clinoptilolite crystallinity structure were noticed. For the ALG-BC, the sharp peaks at 2θ = 20.80, 26.60, 29.40, and 31.00° are associated with the biochar composition [48]**.** For the ALG-CPL composite the peaks at 2θ = 9.90, 11.23, 17.40, 22.34, 28.05, and 30.10° are characteristic of the crystal structure of clinoptilolite [49]. The XRD data indicate clearly that the additives particles are present within the alginate lattice.

Figure 4b presents the ATR/FT-IR spectra of ALG-based adsorbents. The bands in the range of 3000–3600 cm^−1^ were ascribed to the O-H stretching vibrations, while those at about 1586, 1412 correspond to the asymmetric and symmetric −COO stretching vibrations. Additionally, the C-O, C-O-C and C-C stretching vibrations were found at 1172, 1084 and 1014 cm^−1^. After the modification of the alginate structure with biochar, clinoptilolite, lignin, and cellulose, the dominant bands of alginate were retained in the new synthetized alginate composites which were slightly shifted and changed intensity. Many bands in the range of 800–1500 cm^−1^ were characteristic of the clinoptilolite, lignin and cellulose chemical structures. The bands located at 1067, 796 and 469 cm^− 1^ were identified for clinoptilolite and corresponded to the asymmetric Si–O and Al-O stretching vibrations in SiO_4_ or AlO_4_ tetrahedra (1067 cm^−1^), the O–Si–O and O-Al-O stretching vibrations (796 cm^−1^) as well as the Si–O and Al-O bending vibrations (469 cm^−1^) [50]. For lignin, the medium beads found at 1509 cm^−1^ could contributed to the CH deformation (methyl and methylene). The lignin structures in the range of 1100–1300 cm^−1^ are possibly due to the C=O stretching of syringyl and guaiacyl ring [51]. The sharp band at 2899 cm^−1^ was associated with the CH_2_ groups of cellulose. The tiny band found at 1310 cm^−1^ was attributed to the presence of the small amount of C–O stretching vibrations of the syringyl ring units, while the band at 1166 cm^−1^ signifies the C–O–C groups of cellulose [51,52].

The wide-scan XPS spectra of ALG, ALG-BC, ALG-CPL, ALG-L, and ALG-C are illustrated in Figure 4c. The main elemental C1, O1, N1, and Ca2p bands distinguished the alginate-based biosorbents. Additionally, for ALG-BC P2p, Si2p, and Mg2p; for ALG-CPL Si2s, Si2p, Al2s, and Al2p; and for ALG-L S2p signals were recorded. The O1s peaks of all biosorbents at 531.5–534.2 eV corresponded to the O-C-O and C-O-H groups. The peaks at 283.3-289.6 eV were associated with C1s in the C-OH, C-C, C=O, and C-OO^-^ bonds. It was also observed that the peaks at about 348.5 eV corresponded to Ca2p. The XPS results indicated the successful incorporation of additives into the alginate structure. In addition, Fila et al. [36] found a complete disappearance of Ca2p-derived signals after the sorption process of La(III) ions, further confirming the ion exchange mechanism taking place on the alginate sorbents.

The TG analysis in the temperature range 300–1300 K was performed to evaluate the thermal behavior of the alginate-based adsorbents. The curves of the weight percent of five materials (ALG, ALG-BC, ALG-CPL, ALG-L, and ALG-C) with respect to the temperature are illustrated in Figure 4d. At the beginning a similar thermal degradation of biosorbents can be seen. The highest weight of biosorbents (about 40% weight loss) was lost from 450 to 600 K0% of the weight loss of the ALG, ALG-BC, ALG-CPL, ALG-L, and ALG-C samples, observed at 576, 707, 643, 618, and 640 K, respectively. Additionally, when the temperature increased to 1300 K the solid residue weights were 22.65% for ALG, 34.08% for ALG-BC, 30.33% for ALG-CPL, 18.25% for ALG-L, and 28.32% for ALG-C. The obtained TG results suggested that the ALG stability was improved especially after the addition of BC and CPL.

The gradation test results (Figure 4e) and the digital images obtained by optical microscopy (Figure 4f) demonstrated that the mean diameter of the alginate-based adsorbent beads were 1.1 ± 0.005 mm. These studies confirmed that the synthetized adsorbent beads exhibit monodispersity. The ALG, ALG-CPL, ALG-L and ALG-C beads were 99% of 1100 µm in diameter. In the case of ALG-BC beads, their diameter in 95.14% was equal to 1100 µm. The microscopic images clearly show the difference in the color of the obtained alginate adsorbent beads.

### 3.2. Column Studies Results

**Figure 5:**
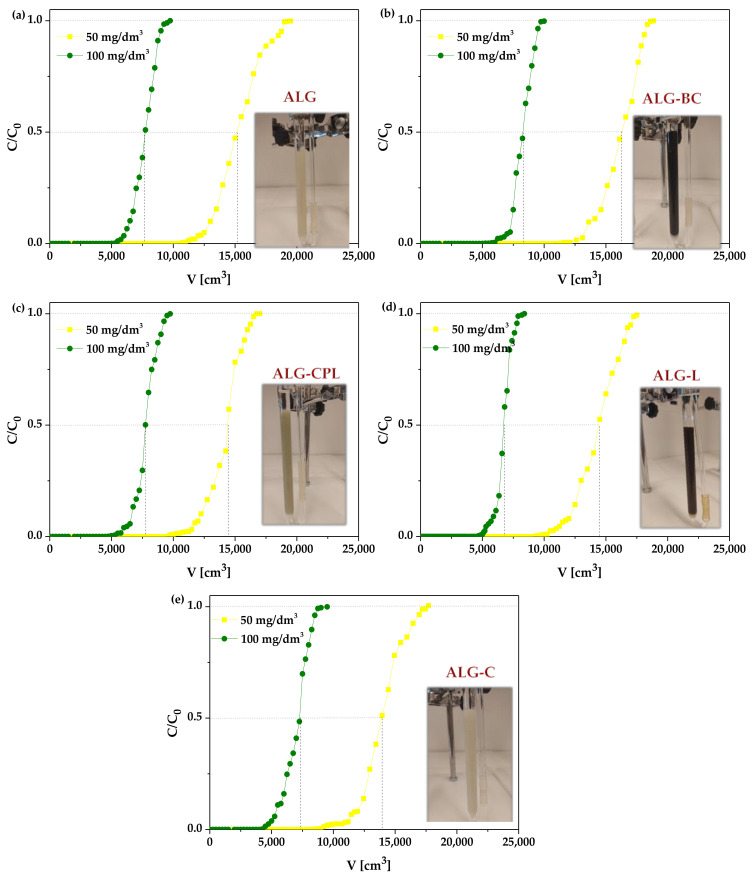
Breakthrough curves of La(III) ions sorption for the alginate-based adsorbents: (**a**) ALG, (**b**) ALG-BC, (**c**) ALG-CPL, (**d**) ALG-L, and (**e**) ALG-C.

To describe the ion exchange process properly, the shape of the breakthrough curve and the adsorbent exchange capacity under the specified conditions must be determined for the dynamic technique. When considering the parameters of the column experiments, it can be seen that lower flow rates, large bed depths, and lower adsorbate concentrations are recommended to achieve the best dynamic adsorption results [53]. According to Donia et al. [54], the breakthrough occurred more quickly at higher flow rates when taking the flow rate into account. This behavior could result from the metal ions short residence time on the column which has a detrimental impact on the interaction process and the metal ions diffusion through the pores of beads. To assess the utility of the alginate-based adsorbents in the column studies, the La(III) ion sorption process was conducted to obtain breakthrough curves. The column experiments involved passing a solution of La(III) ions at the initial concentrations of 50 or 100 mg/dm^3^ at pH = 5.0 through a column filled with 10 cm^3^ of suitable ALG-based adsorbents at the flow rate of 0.6 cm^3^/min. The breakthrough curves (C/C_0_ vs. V) were used to calculate the parameters for the column experiments. The breakthrough curves of the alginate-based adsorbents for removal of La(III) ions with different initial concentrations are shown in Figure 5, and the corresponding parameters from the plotted models and calculations are summarized in Table 2.

The obtained breakthrough curves presented in Figure 5 resemble the typical S-shaped profile created by nearly perfect adsorption systems. The breakthrough curves analysis revealed that the initial solution concentration influences the ion exchange capabilities of the ALG-based adsorbents in terms of La(III) ions. As the initial La(III) concentration increases, the volume of solution required to reach the breakthrough point decreases. At C_0_ = 100 mg/dm^3^, the volume of La(III) ion solution required to saturate the bed is less than at C_0_ = 50 mg/dm^3^. It follows from the above that the breakthrough time was also changed when the initial La(III) concentration increased from 50 to 100 mg/dm^3^. In the case of 50 mg/dm^3^ of La(III) solution the breakthrough point time was achieved after 285 h for ALG, 302 h for ALG-BC, 267 h for ALG-CPL, 260 h for ALG-L, and 222 h for ALG-C. Using 100 mg/dm^3^ of La(III) solution, the breakthrough point time was reached after 138 h for ALG, 139 h for ALG-BC, 132 h for ALG-CPL, 128 h for ALG-L, and 111 h for ALG-C. Besides the column breakthrough point, the time needed to achieve the saturation point is another important parameter. Using 50 mg/dm^3^ of La(III) solution, the saturation point time was reached after 524 h for ALG, 542 h for ALG-BC, 472 h for ALG-CPL, 486 h for ALG-L, and 492 h for ALG-C. On the other hand, for 100 mg/dm^3^ of La(III) solution, the saturation point time was reached after 270 h for ALG, 278 h for ALG-BC, 271 h for ALG-CPL, 233 h for ALG-L, and 264 h for ALG-C.

**Figure 6:**
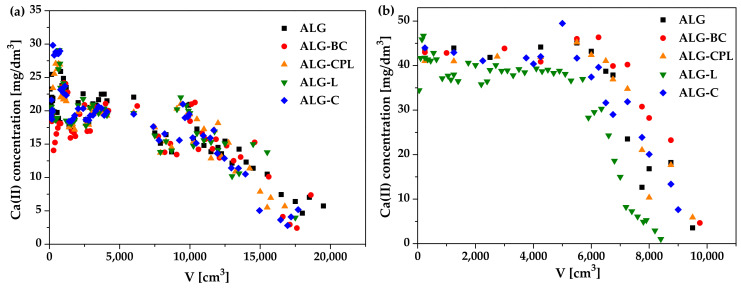

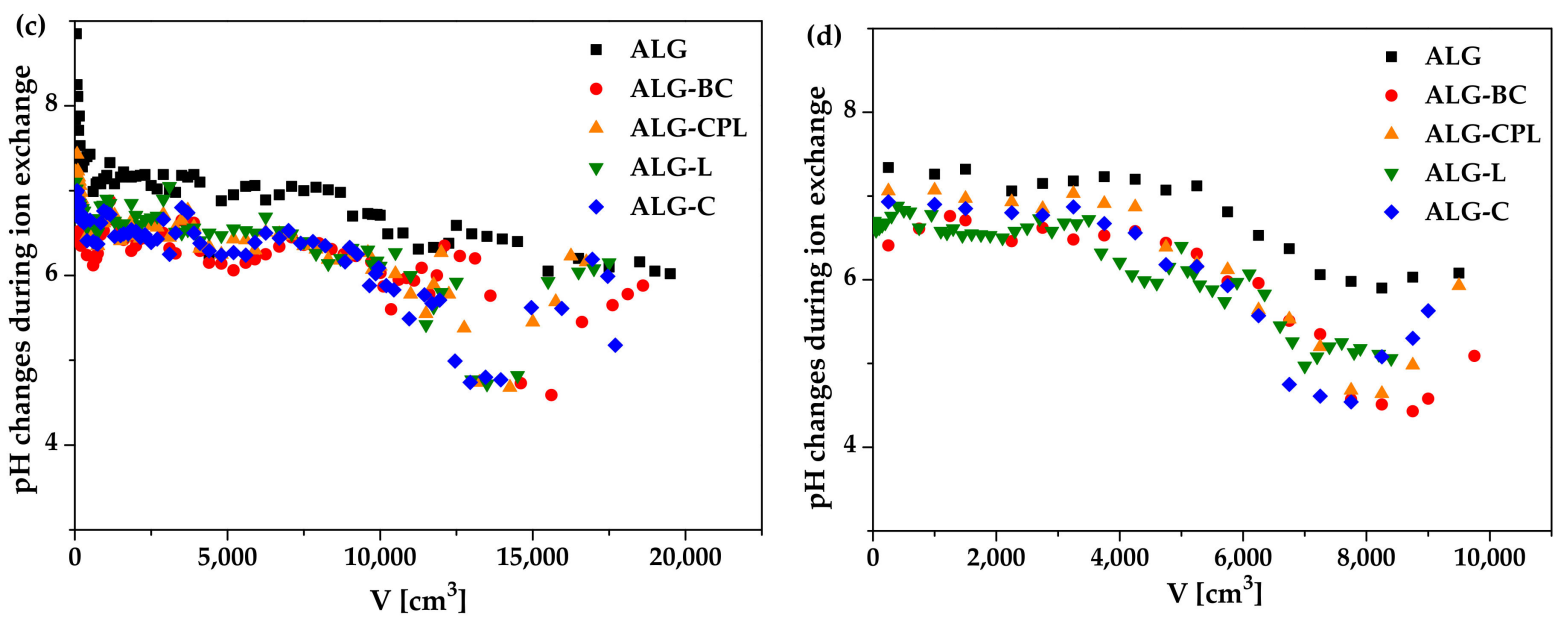
Changes in the Ca(II) ion concentration and solution pH during the ion exchange onto the alginate-based adsorbents for (**a**,**c**) 50 mg/dm^3^ as well as (**b**,**d**) 100 mg/dm^3^ of La(III).

Moreover, during the biosorption column experiments, the Ca(II) ions release profiles and the solution pH changes were determined to understand the biosorption mechanism in the continuous fixed-bed system. The profiles of Ca(II) ions released by ALG, ALG-BC, ALG-CPL, ALG-L, and ALG-C beads as well as pH changes during the ion exchange process are presented in Figure 6.

As follows from Figure 5 and Figure 6a,b, La(III) ions were initially trapped by the alginate-based adsorbent beads close to the column entrance, and then Ca(II) ions were released to the liquid phase. The concentration of Ca(II) ions peaked close to the breakthrough point, whereas that La(III) ions continued to rise. The following drop in the Ca(II) ions concentration reveals the exhaustion of the exchange capacity of the biosorption column. After passing the bed saturation point, the concentration of Ca(II) ions in the solution approaches zero. Figure 6c,d presents the changes in the pH solution during the column process. When the adsorbent bed is gradually saturated with La(III) ions, the pH of the solution changes slightly at first. Once the breakthrough point is reached, the pH of the solution begins to decrease. The above changes indicate that the process of exchanging calcium(II) ions for lanthanum(III) ones takes place.

The breakthrough data for the studied ALG-based adsorbents is given in Table 2. The ion exchange affinities of the various alginate adsorbents clearly differ from one another. The La(III) ion adsorption capacities using 50 mg/dm^3^ of La(III) for alginate, alginate-biochar, alginate-clinoptilolite, alginate-lignin, and alginate-cellulose were 211.08, 233.31, 193.94, 215.45, and 184.02 mg/g, respectively. For 100 mg/dm^3^ of La(III) the adsorption capacities were as follows: 203.15, 239.56, 207.35, 193.22, and 177.06 mg/g for alginate, alginate-biochar, alginate-clinoptilolite, alginate-lignin, and alginate-cellulose, respectively. Comparing the above results with the literature data, the synthesized alginate-based adsorbents are very efficient and promising for column studies. For example, da Costa et al. [22] studied lanthanum(III) biosorption using the sericin/alginate/polyvinyl alcohol beads in the fixed-bed column system. The highest amount of lanthanum biosorption at the saturation was found to be 98.60 mg/g. Texier et al. [55] investigated La(III), Eu(III), and Yb(III) ions removal by *Pseudomonas aeruginosa* immobilized in the polyacrylamide gel. The maximum adsorption capacities were found with 208 µmol/g (28.89 mg/g) for lanthanum, 219 µmol/g (30.42 mg/g) for europium and 192 µmol/g (26.67 mg/g) for ytterbium. Oliviera et al. [56] applied the *Sargassum* sp biomass in the fixed-bed columns for the biosorption and desorption of lanthanum (La(III)) and neodymium (Nd(III)) ions. The maximum biosorption uptake reached 0.57 mmol/g (79.17 mg/g) for La(III) and 0.55 mmol/g (76.40 mg/g) for Nd(III).

Comparing the obtained results for raw alginate, lanthanum(III) was not present in the 50 mg/dm^3^ solution until 10,250 cm^3^ of the solution was passed, while in the 100 mg/dm^3^ solution it began to appear after about 5000 cm^3^. At C_0_ = 50 mg/dm^3^, the volume needed to reach the breakthrough point for alginate-biochar, alginate-clinoptilolite, alginate-lignin, and alginate-cellulose was 10,860, 9600, 9350, and 8000 cm^3^, respectively. For the 100 mg/dm^3^ concentration, the values of the solution volume to reach the breakthrough point were lower in the range of 4000–5000 cm^3^. The saturation point for alginate were achieved after passing 18,600 cm^3^ 50 mg/dm^3^ of La(III) solution. The alginate-biochar composite becomes saturated after passing about 19,500 cm^3^ through the column. In the case of the alginate-clinoptilolite beads, approximately 17,700 cm^3^ of La(III) ions solution was needed for the column saturation. Similar results were obtained for the alginate-lignin composite where 17,500 cm^3^ of La(III) solution was needed for saturation. The lowest volume of the solution (although still very promising) needed to saturate the column was needed for the alginate-cellulose, i.e., 17,000 cm^3^. For the 100 mg/dm^3^ of La(III) solution, the solution volume needed for the columns saturation was lowest and the column breakthrough time was faster.

The total and working exchange capacities as well as the volumetric and mass distribution coefficient values are compared in Table 2. Based on the data, it was found that the initial concentration changing from 50 to 100 mg/dm^3^ causes a change in the parameters q_ec_, C_t_, C_w_, D_g_, and D_v_. Comparing the alginate-biochar to other composites, this adsorbent is characterized by the highest working ion exchange capacity relation to La(III) ions, both at C_0_ 50 and 100 mg/dm^3^, which are 54.99 and 50.90 mg/cm^3^, respectively. The other column parameters were also the highest for this biosorbent. The lowest value of the mentioned parameters was obtained for the alginate-cellulose composite.

Based on the obtained results, the series of affinities of the alginate-based adsorbents for La(III) ions depending on the initial metal solution concentration was as follows: at C_0_ = 50 mg/dm^3^: alginate-biochar (ALG-BC) > alginate-lignin (ALG-L) > alginate (ALG) > alginate-clinoptilolite (ALG-CPL) > alginate-cellulose (ALG-C), and at C_0_ = 100 mg/dm^3^: alginate-biochar (ALG-BC) > alginate (ALG) > alginate-clinoptilolite (ALG-CPL) > alginate-lignin (ALG-L) > alginate-cellulose (ALG-C).

### 3.3. Column Studies Modelling

The breakthrough curves determined in the column tests were used to simulate and predict the adsorption processes. All of the experimental data were matched to the four mathematical models: the Thomas, Adams-Bohart, Yoon-Nelson, and Wolborska, for better comprehension of the column experiments. The calculated column model parameters are presented in Table 3. It should be noted that with the additive change in alginate and an increase in the initial lanthanum(III) concentration, the values of the Thomas, Adams-Bohart, Yoon-Nelson, and Wolborska model parameters changed.

The Thomas model application enables calculation of the Thomas rate constants (k_Th_) and the maximum adsorption capacities (q_0_). The q_0_ values calculated using this model are completely consistent with the experiment at q_ec_ values presented in Table 2. For the Thomas model, the k_Th_ and q_0_ values were changed with the initial La(III) concentration increase as follows: for alginate the k_Th_ parameter increased whereas the value of q_0_ decreased, for alginate-biochar the k_Th_ parameter decreased whereas the value of q_0_ increased, for alginate-clinoptilolite the k_Th_ parameter decreased whereas the value of q_0_ increased, for alginate-lignin the k_Th_ parameter increased whereas the value of q_0_ decreased, and for alginate-cellulose the k_Th_ parameter increased whereas the value of q_0_ decreased. Similarly, da Costa et al. [31] also found lower q_0_ and higher k_Th_ values under the condition of higher initial concentration in the adsorption studies of Cr(III) by the *Sargassum filipendula* algae waste from the alginate extraction. This Thomas model parameters’ dependences were caused because the concentration difference between the metal ions on the adsorbent and the metal ions in the solution is what drives adsorption. It was also noticed that this relation depends on the adsorbents [57,58]. Better column performance was achieved as a result of the greater driving force brought on by the higher concentration of La(III) ions. In the case of alginate-biochar and alginate-clinoptilolite composites, higher influent concentrations increase the adsorption of lanthanum(III) on the column. However, in the case of alginate, alginate-lignin, and alginate-cellulose, lower influent concentrations cause a higher removal percentage of lanthanum(III) in the column sorption system. The R^2^ values determined using this model range from 0.903 to 0.980 indicating that the Thomas column model is appropriate for La(III) adsorption.

Based on the Yoon–Nelson model, the rate constant (k_YN_), the time required to achieve 50% breakthrough time (τ), and the correlation coefficient (R^2^) were determined and are presented in Table 3. The breakthrough times of the Yoon–Nelson model (τ) were reduced but the rate constant (k_YN_) increased with increasing initial La(III) ions concentration. The higher the τ value (highest for ALG-BC), the better the column performance, as similarly reported by Omitola et al. [59]. The R^2^ values were in the range 0.903–0.937 for ALG, 0.938–0.973 for ALG-BC, 0.908–0.964 for ALG-CPL, 0.958–0.980 for ALG-L, and 0.907–0.971 for ALG-C. The above results indicate that the Yoon–Nelson model is also valid for the La(III) ions adsorption by the alginate biosorbents and represents the experimental breakthrough curve accurately.

It should be emphasized that just the initial part of the curve (when C/C_0_ < 0.5) was taken into account when the Adams-Bohart and Wolborska models were applied. The parameters of the column performance, including the maximum adsorption capacities (q_AB_) and the Adams-Bohart rate constant (k_AB_), were calculated using the Adams-Bohart model. For alginate and alginate-lignin adsorbents, the values of k_AB_ increased with an initial La(III) concentration increase, but in the case of the others, it showed decreasing trends. This showed that the overall system kinetics was dominated by external mass transfer in the initial part of adsorption in the column, especially for alginate and alginate-lignin composites [60]. The Adams-Bohart model parameters were not matched sufficiently with the experimental adsorption data. The R^2^ values for this model were in the range 0.773–0.808 for ALG, 0.912–0.916 for ALG-BC, 0.900–0.931 for ALG-CPL, 0.870–0.917 for ALG-L, and 0.674–0.868 for ALG-C. Thus, the best fitting of this model was obtained for the La(III) adsorption on the alginate-biochar, alginate-clinoptilolite, and alginate-lignin composites. In the case of the Wolborska model, the external mass transfer kinetic coefficient (βa) and the maximum amount of adsorbent that can be adsorbed per unit volume (q) were estimated. When the initial La(III) ion concentration increased, the βa coefficient for alginate and alginate-lignin increased, but it decreased for alginate-biochar, alginate-clinoptilolite, and alginate-cellulose. This model is useful in the initial part of the curve at relatively low adsorbate concentrations. External mass transfer was thought to be the primary factor in La(III) adsorption at relatively low concentrations [61]. The increased probability of adsorption due to an increase in La(III) concentration could be the cause of the increase in adsorption capacity (q) for ALG, ALG-BC, and ALG-CPL. The obtained R^2^ values were not particularly high, ranging from 0.773 to 0.931.

In conclusion, it can be stated that the Yoon-Nelson and Thomas models are suitable for the time interval between the breakthrough and saturation points. In both models, higher R^2^ values were obtained compared to those obtained using the Adam-Bohart and Wolborska models. Additionally, the Thomas model assumes that the driving force of adsorption is governed by the PSO kinetics and the best Langmuir fits as observed in the previous papers for the alginate-based adsorbents in the batch experiments [36,37]. Based on the R^2^ values comparison, both the Yoon-Nelson and Thomas models could be applied to predict adsorption performance for the adsorption of La(III) ions in the fixed-bed column. These results are in agreement with the column studies of heavy metal biosorption by alginate and alginate-Spirulina reported by Kőnig-Péter et al. [62] as well as by the nanochitosan/sodium alginate/microcrystalline cellulose beads reported by Vijayalakshmi et al. [63].

**Figure 7:**
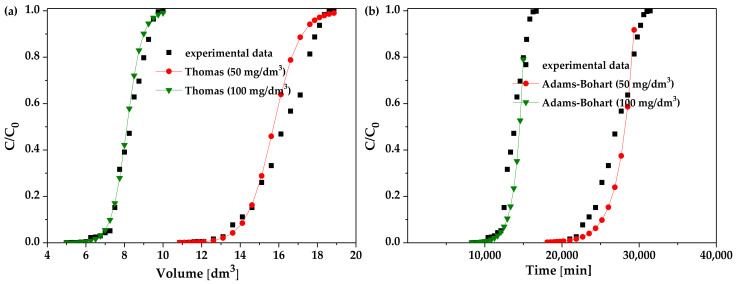

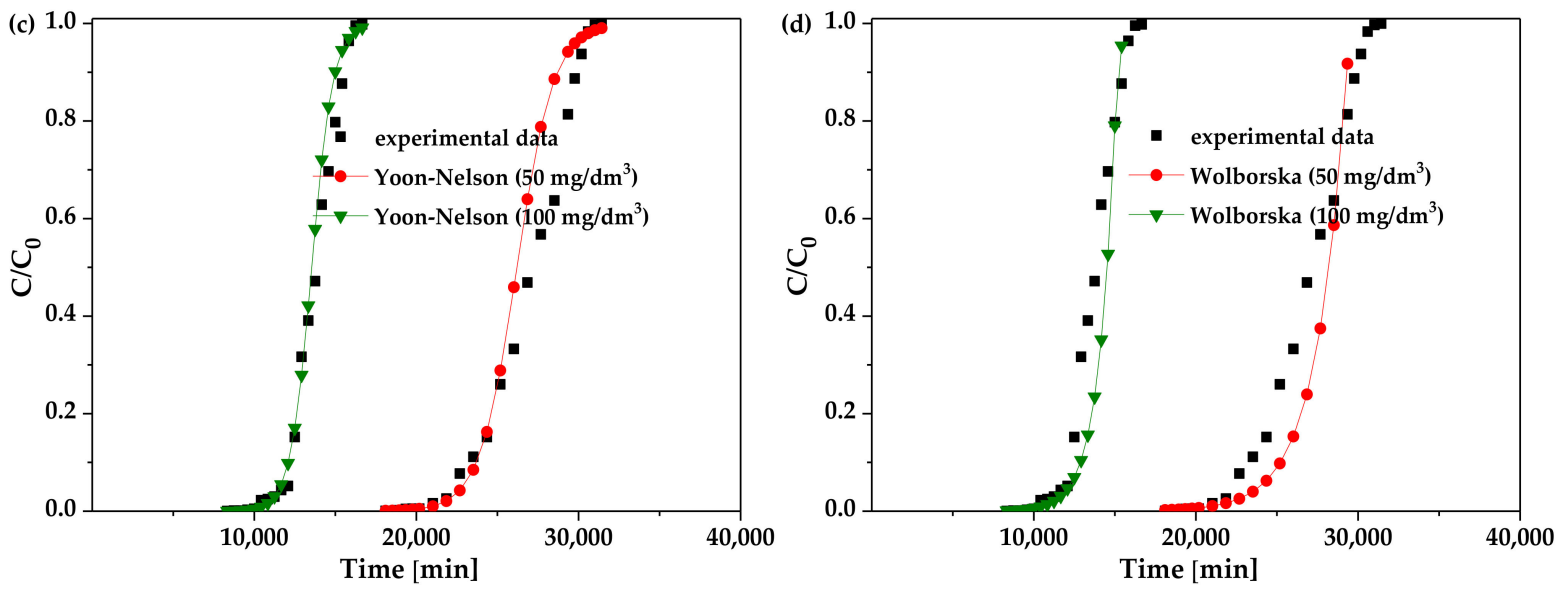
Fitting of nonlinear (**a**) Thomas, (**b**) Adams-Bohart, (**c**) Yoon-Nelson, and (**d**) Wolborska models to the experimental data from the La(III) adsorption in the fixed-bed column studies for the alginate-biochar composite.

For the best alginate-based adsorbent selected in the fixed-bed column studies—the alginate-biochar composite—the nonlinear fitting of the Thomas, Adams-Bohart, Yoon-Nelson, and Wolborska models to the breakthrough curves was estimated and presented in Figure 7. The nonlinear fitting confirmed the obtained dependences.

### 3.4. Desorption Studies

**Figure 8:**
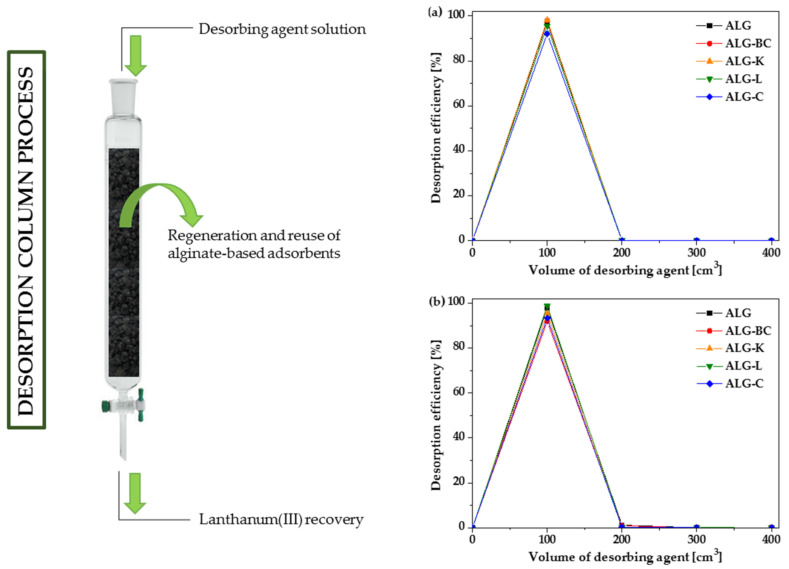
Desorption column results for the alginate-based adsorbents using (**a**) 50 mg/dm^3^ La(III), (**b**) 100 mg/dm^3^ La(III).

Taking into consideration a prospective implementation of the alginate adsorbents on an industrial scale, desorption studies of the prepared beads were carried out. As a desorbing agent, 1 mol/dm^3^ of HCl solution was used to estimate the desorption ability of alginate-based adsorbents. In this study, the desorption of La(III) ions from the ALG-based adsorbent surfaces was examined using the 50 and 100 mg/dm^3^ solution of La(III) ions. When the adsorbents beds in the columns were completely filled by La(III) ions (i.e., when the La(III) ion concentration in the effluent was equal to concentration of the La(III) ion introduced into the column), the desorption process was initiated. The desorption process was conducted by pouring a specific portion of the desorbing agent onto the column and then testing for the presence of La(III) ions in the effluent by ICP-OES. This process went on until no La(III) ions were present in the effluent. The peaks obtained after the desorption process are illustrated in Figure 8.

The results presented in Figure 8 show a high degree of reusability for the ALG-based adsorbents using 1 mol/dm^3^ of HCl solution. The ALG-based adsorbents packed columns showed the maximum desorption of La(III) just after passing 100 cm^3^ of 1 mol/dm^3^ HCl. Figure 7 depicts the desorption process from 50 mg/dm^3^ of La(III) solution onto the alginate-based adsorbents which resulted in the yield of 92.20-98.33%. In the case of 100 mg/dm^3^ La(III) solution, %D ranged from 92.84 to 99.35%. This great reusability is consistent with the static results for the La(III) ion desorption on the alginate-based adsorbents reported in my previous studies [36,37]. As illustrated in Figure 8, the elution of La(III) ions was fast. Complete regeneration of the ALG-based adsorbents from La(III) ions took place after passing 200 cm^3^ of 1 mol/dm^3^ HCl solution. Wu et al. [64] performed effective sorption/desorption cycles for the La(III)-loaded columns filled with the magnetic alginate beads (iron oxide loaded calcium alginate) using 0.05 mol/dm^3^ CaCl_2_ as an eluent. Costa et al. [22] evaluated lanthanum(III) biosorption in the fixed-bed flow-through column using the sericin/alginate/polyvinyl alcohol beads as a natural cation exchanger. The La(III) desorption process was successful after four regeneration cycles, with a recovery rate of more than 95%.

## 4. Conclusions

In this study, the alginate-based adsorbents, i.e., raw calcium alginate, alginate-biochar, alginate-clinoptilolite, alginate-lignin, and alginate-cellulose, were synthetized to evaluate the adsorption effectiveness of La(III) ions in the fixed-bed column studies. The adsorption of lanthanum(III) ions on the above biosorbents was affected by the initial La(III) concentration. The results of the column experiment showed that the ALG-based composite beads are effective adsorbents for the uptake of La(III) from the aqueous acidic medium at pH 5, with the maximum uptake capacity of alginate-biochar equal to 239.56 mg/g. Moreover, the initial lanthanum(III) ion concentration had a significant effect on the obtained breakthrough curves. When the initial La(III) ion concentration increased, the breakthrough time and exhaustion time decreased. Based on the R^2^ value comparison of the column mathematical models, the Yoon–Nelson and Thomas models were found to be appropriate for the description of the breakthrough curve data in the fixed-bed column, whereas the Bohart–Adams and Wolborska models did not match very well. The exhausted alginate-based adsorbent beads can be easily regenerated by treating them with 1 mol/dm^3^ HCl. The column adsorption studies show that alginate-based adsorbents are highly efficient natural cation exchangers for lanthanum(III) ion recovery.

## Figures and Tables

**Table 1:** Comparison of the ALG-based adsorbents properties.

Biosorbents	Appearance	Functional Groups	pH_pzc_	S_BET_ [m^2^/g]	D [nm]	V_total_ [cm^3^/g]
Calcium alginate	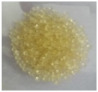	Carboxyl, hydroxyl	6.14	4.7	3.14	0.77 × 10^−2^
Alginate-biochar	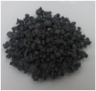	Carboxyl, hydroxyl, phenolic, lactonic, aromatic and heterocyclic carbons	7.68	9.3	3.51	1.62 × 10^−2^
Alginate-clinoptilolite	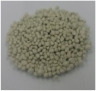	Carboxyl, hydroxyl, hydrated aluminosilicates (Na_6_Al_6_Si_30_O_72_·24H_2_O)	6.27	6.7	3.14	2.54 × 10^−2^
Alginate-lignin	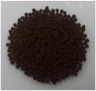	Carboxyl, hydroxyl, methoxyl, carbonyl, benzyl alcohol	5.85	8.0	2.98	1.07 × 10^−2^
Alginate-cellulose	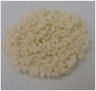	Carboxyl, hydroxyl, carbonyl, ether	5.67	9.8	3.32	1.65 × 10^−2^

**Table 2:** Dynamic studies parameters of La(III) ions adsorption onto the alginate-based adsorbents (pH = 5.00 ± 0.05).

Adsorbent	C_0_ [mg/dm^3^]	U [cm^3^]	Ū [cm^3^]	q_ec_ [mg/g]	C_t_ [mg/cm^3^]	C_t integration_ [mg/cm^3^]	C_w_ [mg/cm^3^]	D_g_	D_v_
**ALG**	50	10,250	15,200	211.08	76.97	76.09	51.90	4215.20	1519.56
	100	5000	7750	203.15	75.02	73.24	48.40	2148.60	774.56
**ALG-BC**	50	10,860	16,360	233.31	82.84	80.63	54.99	4732.59	1635.56
	100	5000	8300	239.56	84.50	82.79	50.90	2400.38	829.56
**ALG-CPL**	50	9600	14,400	193.94	72.92	72.81	48.61	3834.28	1439.56
	100	4750	7750	207.35	78.90	77.85	48.36	2063.05	774.56
**ALG-L**	50	9350	14,450	215.45	73.17	71.48	47.35	4354.30	1444.56
	100	4600	6700	193.22	64.86	64.10	44.53	2018.24	669.56
**ALG-C**	50	8000	13,900	184.02	70.39	69.32	40.51	3688.62	1389.56
	100	4000	7350	177.06	71.15	66.70	38.72	1949.91	734.56

**Table 3:** Breakthrough fitting models results for the La(III) ions adsorption on the alginate-based adsorbents.

Model		Thomas			Adams–Bohart		
Adsorbent	C_0_ [mg/dm^3^]	k_Th_ × 10^−3^ [cm^3^/mg × min]	q_0_ [mg/g]	R^2^	k_AB_ × 10^−6^ [dm^3^/mg × min]	q_AB_ [mg/dm^3^]	R^2^
ALG	50	14.31	215.50	0.937	8.29	94,000.02	0.773
	100	16.69	202.02	0.903	8.59	98,313.82	0.808
ALG-BC	50	17.49	230.35	0.938	10.61	95,131.51	0.912
	100	14.19	239.35	0.973	9.55	98,783.35	0.916
ALG-CPL	50	16.71	185.40	0.908	9.18	89,257.08	0.931
	100	13.45	206.97	0.964	7.70	99,745.01	0.900
ALG-L	50	13.34	216.24	0.980	8.10	95,444.54	0.917
	100	19.45	191.96	0.958	10.51	85,485.15	0.870
ALG-C	50	13.53	185.91	0.971	8.63	89,757.75	0.868
	100	14.40	177.36	0.907	8.02	87,596.47	0.674
		Yoon–Nelson			Wolborska		
Adsorbent	C_0_ [mg/dm^3^]	k_YN_ × 10^−3^ [1/min]	*τ* [min]	R^2^	β_a_ [1/min]	q [mg/dm^3^]	R^2^
ALG	50	0.72	25,569.10	0.937	0.862	94,000.02	0.773
	100	1.62	12,538.36	0.903	0.731	98,313.82	0.808
ALG-BC	50	0.89	26,201.99	0.938	0.834	95,131.51	0.912
	100	1.52	13,542.03	0.973	0.951	98,783.35	0.916
ALG-CPL	50	0.85	22,910.02	0.908	0.819	89,257.08	0.931
	100	1.37	12,721.26	0.964	0.718	99,745.01	0.900
ALG-L	50	0.67	23,611.27	0.980	0.739	95,444.54	0.917
	100	1.88	10,964.27	0.958	0.924	85,485.15	0.870
ALG-C	50	0.68	23,051.38	0.971	0.768	89,757.75	0.868
	100	1.39	11,502.97	0.907	0.548	87,596.47	0.827

## Data Availability

Data are included in the paper.

## References

[1] Wang L.M., Lin Q., Yue L.J., Liu L., Guo F., Wang F.M. (2008). Study of application of rare earth elements in advanced low alloy steels. J. Alloys Compd..

[2] Zhang S., Saji S.E., Yin Z., Zhang H., Du Y., Yan C.H. (2021). Rare-Earth Incorporated Alloy Catalysts: Synthesis, Properties, and Applications. Adv. Mater..

[3] Alam M.A., Zuga L., Pecht M.G. (2012). Economics of rare earth elements in ceramic capacitors. Ceram. Int..

[4] Neacsu I.A., Stoica A.E., Vasile B.S., Andronescu E. (2019). Luminescent hydroxyapatite doped with rare earth elements for biomedical applications. Nanomaterials.

[5] Li H., Wang P., Lin G., Huang J. (2021). The role of rare earth elements in biodegradable metals: A review. Acta Biomater..

[6] Townley H.E. (2013). Applications of the Rare Earth Elements in Cancer Imaging and Therapy. Curr. Nanosci..

[7] Roshanfekr Rad L., Anbia M. (2021). Zeolite-based composites for the adsorption of toxic matters from water: A review. J. Environ. Chem. Eng..

[8] Ferreira L.M., Melo R.R. (2021). de Use of Activated Charcoal As Bio-Adsorbent for Treament of Residual Waters: A Review. Nativa.

[9] Ghosh N., Das S., Biswas G., Haldar P.K. (2022). Review on some metal oxide nanoparticles as effective adsorbent in wastewater treatment. Water Sci. Technol..

[10] Ferhan C., Ozgur A. (2011). Activated Carbon for Water and Wastewater Treatment: Integration ofAdsorption and Biological Treatment.

[11] Jjagwe J., Olupot P.W., Menya E., Kalibbala H.M. (2021). Synthesis and application of granular activated carbon from biomass waste materials for water treatment: A review. J. Bioresour. Bioprod..

[12] De Freitas G.R., da Silva M.G.C., Vieira M.G.A. (2019). Biosorption technology for removal of toxic metals: A review of commercial biosorbents and patents. Environ. Sci. Pollut. Res..

[13] Wang B., Wan Y., Zheng Y., Lee X., Liu T., Yu Z., Huang J., Ok Y.S., Chen J., Gao B. (2019). Alginate-based composites for environmental applications: A critical review. Crit. Rev. Environ. Sci. Technol..

[14] Fuks L., Oszczak-Nowińska A. (2021). Sorption of selected radionuclides from liquid radioactive waste by sorbents of biological origin: The alkaline earth alginates. Nukleonika.

[15] Sutirman Z.A., Sanagi M.M., Wan Aini W.I. (2021). Alginate-based adsorbents for removal of metal ions and radionuclides from aqueous solutions: A review. Int. J. Biol. Macromol..

[16] Gao X., Guo C., Hao J., Zhao Z., Long H., Li M. (2020). Adsorption of heavy metal ions by sodium alginate based adsorbent-a review and new perspectives. Int. J. Biol. Macromol..

[17] Li S.S., Song Y.L., Yang H.R., Da An Q., Xiao Z.Y., Zhai S.R. (2020). Modifying alginate beads using polycarboxyl component for enhanced metal ions removal. Int. J. Biol. Macromol..

[18] Saad E.M., Elshaarawy R.F., Mahmoud S.A., El-Moselhy K.M. (2021). New *Ulva lactuca* algae based chitosan bio-composites for bioremediation of Cd(II) ions. J. Bioresour. Bioprod..

[19] Xu X., Wang B., Tang H., Jin Z., Mao Y., Huang T. (2020). Removal of phosphate from wastewater by modified bentonite entrapped in Ca-alginate beads. J. Environ. Manage..

[20] Shehzad H., Ahmed E., Sharif A., Farooqi Z.H., Din M.I., Begum R., Liu Z., Zhou L., Ouyang J., Irfan A. (2022). Modified alginate-chitosan-TiO_2_ composites for adsorptive removal of Ni(II) ions from aqueous medium. Int. J. Biol. Macromol..

[21] Abd-Elhamid A.I., Elgoud E.M.A., Aly H.F. (2022). Alginate modified graphene oxide for rapid and effective sorption of some heavy metal ions from an aqueous solution. Cellulose.

[22] Da Costa T.B., da Silva M.G.C., Vieira M.G.A. (2021). Biosorption of lanthanum using sericin/alginate/polyvinyl alcohol beads as a natural cation exchanger in a continuous fixed-bed column system. Colloids Surf. A Physicochem. Eng. Asp..

[23] Benettayeb A., Guibal E., Morsli A., Kessas R. (2017). Chemical modification of alginate for enhanced sorption of Cd(II), Cu(II) and Pb(II). Chem. Eng. J..

[24] Campos N.F., Sales D.C.S., Rodríguez-Díaz J.M., Barbosa C.M.B.M., Duarte M.M.M.B. (2022). Adsorption of naphthenic acids on peanut shell activated carbon: Batch and fixed-bed column study of the process. Chem. Eng. Res. Des..

[25] Fernández-Andrade K.J., González-Vargas M.C., Rodríguez-Rico I.L., Ruiz-Reyes E., Quiroz-Fernández L.S., Baquerizo-Crespo R.J., Rodríguez-Díaz J.M. (2022). Evaluation of mass transfer in packed column for competitive adsorption of Tartrazine and brilliant blue FCF: A statistical analysis. Results Eng..

[26] Bo S., Luo J., An Q., Xiao Z., Wang H., Cai W., Zhai S., Li Z. (2020). Efficiently selective adsorption of Pb(II) with functionalized alginate-based adsorbent in batch/column systems: Mechanism and application simulation. J. Clean. Prod..

[27] Patel H. (2019). Fixed-bed column adsorption study: A comprehensive review. Appl. Water Sci..

[28] Ge Y., Cui X., Liao C., Li Z. (2017). Facile fabrication of green geopolymer/alginate hybrid spheres for efficient removal of Cu(II) in water: Batch and column studies. Chem. Eng. J..

[29] Rivas S.C.M., Corral R.I.A., Félix M. (2019). del C. F.; Rubio, A.R.I.; Moreno, L.V.; Montfort, G.R.C. Removal of cadmium from aqueous solutions by *Saccharomyces cerevisiae*-alginate system. Materials.

[30] Aftab K., Akhtar K., Jabbar A. (2014). Batch and column study for Pb-II remediation from industrial effluents using glutaraldehyde-alginate-fungi biocomposites. Ecol. Eng..

[31] Da Costa T.B., da Silva T.L., Costa C.S.D., da Silva M.G.C., Vieira M.G.A. (2022). Chromium adsorption using *Sargassum filipendula* algae waste from alginate extraction: Batch and fixed-bed column studies. Chem. Eng. J. Adv..

[32] Agrawal P., Bajpai A.K. (2011). Biosorption of chromium(VI) ions from aqueous solutions by iron oxide-impregnated alginate nanocomposites: Batch and column studies. Toxicol. Environ. Chem..

[33] Wang W., Fan M., Ni J., Peng W., Cao Y., Li H., Huang Y., Fan G., Zhao Y., Song S. (2022). Efficient dye removal using fixed-bed process based on porous montmorillonite nanosheet/poly(acrylamide-co-acrylic acid)/sodium alginate hydrogel beads. Appl. Clay Sci..

[34] Vahidhabanu S., Karuppasamy D., Adeogun A.I., Babu B.R. (2017). Impregnation of zinc oxide modified clay over alginate beads: A novel material for the effective removal of congo red from wastewater. RSC Adv..

[35] Verduzco-Navarro I.P., Jasso-Gastinel C.F., Rios-Donato N., Mendizábal E. (2020). Red dye 40 removal by fixed-bed columns packed with alginate-chitosan sulfate hydrogels. Rev. Mex. Ing. Química.

[36] Fila D., Hubicki Z., Kołodyńska D. (2022). Applicability of new sustainable and efficient alginate-based composites for critical raw materials recovery: General composites fabrication optimization and adsorption performance evaluation. Chem. Eng. J..

[37] Fila D., Hubicki Z., Kołodyńska D. (2022). Fabrication, characterization, and evaluation of an alginate-lignin composite for rare-earth elements recovery. Materials.

[38] Lopez-Ramon M.V., Stoeckli F., Moreno-Castilla C., Carrasco-Marin F. (1999). On the characterization of acidic and basic surface sites on carbons by various techniques. Carbon.

[39] Bohart G.S., Adams E.Q. (1920). Some aspects of the behavior of charcoal with respect to chlorine. J. Am. Chem. Soc..

[40] Parimelazhagan V., Jeppu G., Rampal N. (2022). Continuous fixed-bed column studies on congo red dye adsorption-desorption using free and immobilized *Nelumbo nucifera* leaf adsorbent. Polymers.

[41] Thomas H.C. (1944). Heterogeneous ion exchange in a flowing system. J. Am. Chem. Soc..

[42] Han R., Wang Y., Zou W., Wang Y., Shi J. (2007). Comparison of linear and nonlinear analysis in estimating the Thomas model parameters for methylene blue adsorption onto natural zeolite in fixed-bed column. J. Hazard. Mater..

[43] Yoon Y.H., Nelson J.H. (1984). Application of gas adsorption kinetics I. A theoretical model for respirator cartridge service life. Am. Ind. Hyg. Assoc. J..

[44] Wolborska A. (1989). Adsorption on activated carbon of p-nitrophenol from aqueous solution. Water Res..

[45] Satya A., Harimawan A., Haryani G.S., Johir M.A.H., Vigneswaran S., Ngo H.H., Setiadi T. (2020). Batch study of cadmium biosorption by carbon dioxide enriched *Aphanothece* sp. dried biomass. Water.

[46] Ighalo J.O., Adeniyi A.G. (2020). Adsorption of pollutants by plant bark derived adsorbents: An empirical review. J. Water Process Eng..

[47] Gong J., Li J., Xu J., Xiang Z., Mo L. (2017). Research on cellulose nanocrystals produced from cellulose sources with various polymorphs. RSC Adv..

[48] Li C., Huang Q., Zhang H., Wang Q., Xue R., Guo G., Hu J., Li T., Wang J., Hu S. (2021). Characterization of biochars produced by co-pyrolysis of hami melon (*Cantaloupes*) straw mixed with polypropylene and their adsorption properties of cadmium. Int. J. Environ. Res. Public Health.

[49] Güngör D., Özen S. (2021). Development and characterization of clinoptilolite-, mordenite-, and analcime-based geopolymers: A comparative study. Case Stud. Constr. Mater..

[50] Zendelska A., Golomeova M., Lisichkov K., Kuvendziev S. (2018). Characterization and application of clinoptilolite for removal of heavy metal ions from water resources. Geol. Maced..

[51] Md Salim R., Asik J., Sarjadi M.S. (2021). Chemical functional groups of extractives, cellulose and lignin extracted from native *Leucaena leucocephala* bark. Wood Sci. Technol..

[52] Reddy K.O., Ashok B., Reddy K.R.N., Feng Y.E., Zhang J., Rajulu A.V. (2014). Extraction and Characterization of Novel Lignocellulosic Fibers from *Thespesia Lampas* Plant. Int. J. Polym. Anal. Charact..

[53] Rusu L., Grigoraș C.G., Simion A.I., Suceveanu E.M., Dediu Botezatu A.V., Harja M. (2022). Biosorptive Removal of Ethacridine Lactate from Aqueous Solutions by *Saccharomyces pastorianus* Residual Biomass/Calcium Alginate Composite Beads: Fixed-Bed Column Study. Materials.

[54] Donia A.M., Atia A.A., Elwakeel K.Z. (2008). Selective separation of mercury(II) using magnetic chitosan resin modified with Schiff’s base derived from thiourea and glutaraldehyde. J. Hazard. Mater..

[55] Texier A.C., Andrès Y., Faur-Brasquet C., Le Cloirec P. (2002). Fixed-bed study for lanthanide (La, Eu, Yb) ions removal from aqueous solutions by immobilized *Pseudomonas aeruginosa*: Experimental data and modelization. Chemosphere.

[56] Oliveira R.C., Guibal E., Garcia O. (2012). Biosorption and desorption of lanthanum(III) and neodymium(III) in fixed-bed columns with *Sargassum* sp.: Perspectives for separation of rare earth metals. Biotechnol. Prog..

[57] Han R., Wang Y., Zhao X., Wang Y., Xie F., Cheng J., Tang M. (2009). Adsorption of methylene blue by phoenix tree leaf powder in a fixed-bed column: Experiments and prediction of breakthrough curves. Desalination.

[58] Mekonnen D.T., Alemayehu E., Lennartz B. (2021). Fixed-bed column technique for the removal of phosphate from water using leftover coal. Materials.

[59] Omitola O.B., Abonyi M.N., Akpomie K.G., Dawodu F.A. (2022). Adams-Bohart, Yoon-Nelson, and Thomas modeling of the fix-bed continuous column adsorption of amoxicillin onto silver nanoparticle-maize leaf composite. Appl. Water Sci..

[60] Aksu Z., Gönen F. (2004). Biosorption of phenol by immobilized activated sludge in a continuous packed bed: Prediction of breakthrough curves. Process Biochem..

[61] Anbazhagan S., Thiruvengadam V., Kulanthai K. (2020). Adaptive neuro-fuzzy inference system and artificial neural network modeling for the adsorption of methylene blue by novel adsorbent in a fixed-bed column method. Iran. J. Chem. Chem. Eng..

[62] Kőnig-Péter A., Csudai C., Felinger A., Kilár F., Pernyeszi T. (2016). Column studies of heavy metal biosorption by immobilized *Spirulina platensis*-maxima cells. Desalin. Water Treat..

[63] Vijayalakshmi K., Sangeetha K., Sudha P.N. (2017). Analysis of packed bed adsorption column with nanochitosan/sodium alginate/microcrystalline cellulose bead for copper(II) removal from aqueous solution. J. Pharm..

[64] Wu D., Zhao J., Zhang L., Wu Q., Yang Y. (2010). Lanthanum adsorption using iron oxide loaded calcium alginate beads. Hydrometallurgy.

